# Safinamide as an adjunct to levodopa monotherapy in Asian patients with Parkinson’s disease experiencing early wearing-off: a pooled analysis of the J-SILVER and KEEP studies

**DOI:** 10.3389/fneur.2025.1591664

**Published:** 2025-06-02

**Authors:** Noriko Nishikawa, Do-Young Kwon, Yuki Kogo, Taku Hatano, Jin Whan Cho, Chizuru Kobayashi, Hiroyuki Shiiba, JiEun Kim, Takayuki Ishida, Jong Sam Baik, Nobutaka Hattori

**Affiliations:** ^1^Department of Neurology, Faculty of Medicine, Juntendo University, Tokyo, Japan; ^2^Department of Neurology, College of Medicine, Korea University Ansan Hospital, Ansan, Republic of Korea; ^3^Medical Headquarters, Eisai Co. Ltd., Tokyo, Japan; ^4^Department of Neurology, Samsung Medical Center, Sungkyunkwan University School of Medicine, Seoul, Republic of Korea; ^5^Deep Human Biology Learning, Eisai Co., Ltd., Tokyo, Japan; ^6^Department of Medical, Eisai Korea Inc., Seoul, Republic of Korea; ^7^Department of Neurology, Inje University Sanggye Paik Hospital, Seoul, Republic of Korea

**Keywords:** Parkinson’s disease, MAO-B inhibitor, safinamide, sodium channel blocker, early wearing-off, elderly

## Abstract

**Background:**

Limited trials are evaluating the efficacy of monoamine oxidase B inhibitors as an adjunct to levodopa monotherapy for early wearing-off in Parkinson’s disease (PD). We evaluated the efficacy and safety of safinamide in patients with fluctuating PD treated with levodopa monotherapy.

**Methods:**

This pooled analysis used data from the J-SILVER and KEEP studies and targeted patients with PD experiencing wearing-off who received safinamide as adjunct to levodopa monotherapy. Efficacy endpoints were mean changes in 39-item Parkinson’s Disease Questionnaire (PDQ-39), Movement Disorder Society-Unified Parkinson’s Disease Rating Scale (MDS-UPDRS) Parts III and IV, and daily OFF time at 18 weeks of treatment.

**Results:**

Of 54 patients (J-SILVER, *N* = 24; KEEP, *N* = 30), 41 completed the studies. Although not statistically significant, the change in PDQ-39 Summary Index exceeded the minimal clinical important difference (mean [standard deviation (SD)]: −2.2 [7.5], *p* = 0.094) at Week 18. Significant improvements in MDS-UPDRS Parts III and IV scores and daily OFF time were observed at Week 18 from baseline (mean [SD]: −2.8 [8.5]; *p* = 0.043, −1.3 [2.7]; *p* = 0.004, and −1.2 [3.5] hours; *p* = 0.041, respectively). Adverse events occurred in 24 patients (43.6%) and adverse drug reactions (ADRs) occurred in 12 patients (21.8%). ADRs with an incidence ≥5% were dyskinesia (3 events, 5.5%). In subgroup analyses, improvements in PDQ-39 Summary Index and MDS-UPDRS Parts III and IV were significant in patients aged ≥75 years (*p* = 0.039, *p* = 0.029, and *p* = 0.025, respectively).

**Conclusion:**

Safinamide as an adjunct to levodopa monotherapy was effective for early wearing-off without any new tolerability concerns. Safinamide was particularly beneficial in elderly patients.

## 1 Introduction

Parkinson’s disease (PD) is a progressive movement disorder characterized by a loss of dopamine neurons in the substantia nigra pars compacta of the midbrain (1). Levodopa remains the gold standard for the treatment of PD and is currently the first choice in most patients with PD with respect to controlling motor symptoms and safety (2). A retrospective observational study using insurance claims databases that investigated the real-life treatment landscape in Japan has shown that the duration of levodopa monotherapy is longer as the age of onset increases (3), suggesting that the use of levodopa monotherapy will be more common globally due to the aging population. However, sustained and high doses of levodopa lead to motor complications such as wearing-off and dyskinesia (4). The development of wearing-off is an important point in time to consider the next treatment strategy. When patients experience wearing-off, the dose or number of administrations of levodopa may be adjusted, or levodopa adjunctive drugs such as a dopamine agonist (DA), monoamine oxidase B (MAO-B) inhibitor, or catechol-O-methyltransferase (COMT) inhibitor are added to avoid dyskinesia. Since frequent levodopa dosing can lead to poor treatment adherence, adjunctive drugs are one option to overcome this challenge (5, 6). Therefore, determination of which drug should be added next to levodopa should be a focus; however, there are few reports currently examining the efficacy and safety of levodopa adjuncts.

Safinamide is a selective and reversible MAO-B inhibitor for the treatment of PD, and its efficacy for the treatment of patients experiencing wearing-off has been established (7–12). However, the participants were aged in their 60s, which is younger than patients typically seen in clinical practice. In addition, most patients enrolled in clinical trials thus far had advanced-stage wearing-off and were being treated concomitantly with DAs and COMT inhibitors (7–12). Only one publication, a post-hoc analysis of clinical trials, reported that the combination of levodopa with safinamide improved wearing-off (13).

The J-SILVER and KEEP studies were conducted to reveal the efficacy and safety of safinamide in clinical practice for patients with PD and wearing-off (14, 15). The J-SILVER study was conducted in Japan as an observational study to evaluate the efficacy and safety of safinamide as an adjunct to levodopa monotherapy in patients with PD experiencing wearing-off (14). The prospective, interventional KEEP study in Korea investigated the efficacy and safety of safinamide as an adjunct to levodopa alone or in combination with a DA (15). Both studies evaluated the efficacy at 18 weeks by the patient-reported outcome, 39-item Parkinson’s Disease Questionnaire (PDQ-39), and the physician-rated scale, Movement Disorder Society-Unified Parkinson’s Disease Rating Scale (MDS-UPDRS).

To characterize the efficacy of safinamide added to levodopa monotherapy in patients with PD experiencing wearing-off, we performed a pooled analysis using a subset of patients from both the J-SILVER and KEEP studies. Subgroup analyses also evaluated patient characteristics that were predictive of efficacy, including sex, age, disease duration, and daily levodopa dose at baseline. In addition, path analysis was also performed to identify factors associated with quality of life (QOL) improvements.

## 2 Materials and methods

### 2.1 Study design

This was a pooled analysis using data from two clinical studies, the J-SILVER (UMIN: UMIN000044341) and KEEP (https://clinicaltrials.gov/: NCT05312632) studies (14, 15). Only patients who received safinamide as an adjunct to levodopa monotherapy were included in the analyses; patients receiving concomitant DAs in the KEEP study were excluded.

The J-SILVER study was an 18-week prospective, observational study conducted in Japan (14). Patients aged ≥20 years who were diagnosed with PD based on the International Parkinson and Movement Disorder Society (MDS) criteria were enrolled. Patients treated with an oral levodopa monotherapy and had wearing-off with a predictable OFF time were eligible. Other levodopa adjuncts such as DAs or COMT inhibitors were prohibited in this study. Safinamide was initiated at a dose of 50 mg/day and was increased to 100 mg/day at the physician’s discretion if there were no tolerability issues.

The KEEP study was an 18-week prospective, interventional study conducted in Korea (15). All patients met MDS diagnostic criteria for PD. Patients were eligible if they were taking levodopa at least three times per day at stable dose for ≥4 weeks before the screening period and had at least 1.5 h of OFF time. DAs were permitted during each study provided there were no changes in dosage. Patients taking COMT inhibitors or other MAO-B inhibitors were excluded. Patients with cognitive impairment were also excluded. Safinamide was initiated at a dose of 50 mg/day for 2 weeks, and all patients received safinamide 100 mg/day after 2 weeks if there were no tolerability issues.

### 2.2 Outcomes

QOL was assessed using the PDQ-39, which is a patient-reported outcome. Motor symptoms and motor complications were assessed using the MDS-UPDRS Parts III and IV, respectively. Mean daily OFF time was assessed using symptom diaries. Pain was assessed using the King’s Parkinson Pain Scale (KPPS). Changes from baseline to Week 18, which were common to both studies, were assessed as efficacy outcomes.

Subgroup analyses were performed according to sex, age, disease duration, and daily levodopa dose at baseline. Patients were divided into two groups based on the median disease duration and daily levodopa dose.

The incidences of adverse events (AEs) and adverse drug reactions (ADRs) were also evaluated as safety outcomes.

### 2.3 Statistical analysis

Analyses were performed using R (version 4.1.0 or higher) and Python (3.10.12). The full analysis set (FAS) comprised all patients who received at least one dose of safinamide and had at least one post-treatment efficacy evaluation available and was used for all efficacy analyses. Missing data were not imputed for each endpoint, and changes from baseline to Week 18 were calculated for complete cases that could be evaluated at both baseline and Week 18. The safety analysis set comprised all patients who received at least one dose of study drug and had at least one post-treatment safety evaluation available and was used for all safety analyses.

Summary statistics, including mean and standard deviation (SD), were used for continuous data, with 95% confidence intervals (CIs) calculated for means, and the number of cases, frequency, and proportion calculated for categorical data.

Continuous data were tested using paired *t*-test, with a two-sided significance level of 5% and a confidence coefficient of 95%.

The incident numbers and proportions of AEs, ADRs, and each AE and ADR were calculated.

### 2.4 Path analysis

The analysis was applied to the FAS. Missing data were imputed using multiple imputation. To investigate the relationship between variables at baseline or changes at Week 18, models were assumed using the observed values or changes in the PDQ-39 Summary Index, MDS-UPDRS Part III and Part IV, and KPPS total score. Standardized estimates were used. Details of the analysis are provided in the Supplement (Supplementary Methods).

## 3 Results

### 3.1 Patient disposition and characteristics

A total of 54 patients were included in the FAS, including 24 patients from the J-SILVER study and 30 patients from the KEEP study. Of these, 41 patients completed treatment (Supplementary Figure 1). The mean age was 69.9 years (min, max: 49, 84), the mean disease duration was 5.0 years, and the mean daily levodopa dose at baseline was 497.8 mg. The mean modified Hoehn & Yahr stage during ON state was 2.3, and the mean MDS-UPDRS Part III score was 23.7 (Table 1).

**Table 1:** Baseline characteristics.

Baseline characteristics	*N* = 54
Female, *n* (%)	32 (59.3)
Age, years, mean (SD)	69.9 (9.2)
Duration of PD, years, mean (SD)	5.0 (3.0)
Daily dose of levodopa, mg/day, mean (SD)	497.8 (188.9)
Modified Hoehn & Yahr stage (ON state), mean (SD) (*n* = 51)	2.3 (0.8)
Daily OFF time, hours, mean (SD) (*n* = 41)	5.7 (3.3)
MDS-UPDRS Part III (ON state), mean (SD) (*n* = 40)	23.7 (11.2)
MDS-UPDRS Part IV, mean (SD) (*n* = 38)	5.1 (2.1)
PDQ-39 Summary Index, mean (SD) (*n* = 36)	23.3 (13.0)
KPPS total score, mean (SD) (*n* = 39)	6.8 (8.4)
Maximum dose of safinamide during the studies	
50 mg/day, *n* (%)	16 (29.6)
100 mg/day, *n* (%)	38 (70.4)

KPPS, King’s Parkinson’s Pain Scale; MDS-UPDRS, Movement Disorder Society-Unified Parkinson’s Disease Rating Scale; PD, Parkinson’s disease; PDQ-39, 39-item Parkinson’s Disease Questionnaire; SD, standard deviation.

### 3.2 Efficacy outcomes

**Figure 1:**
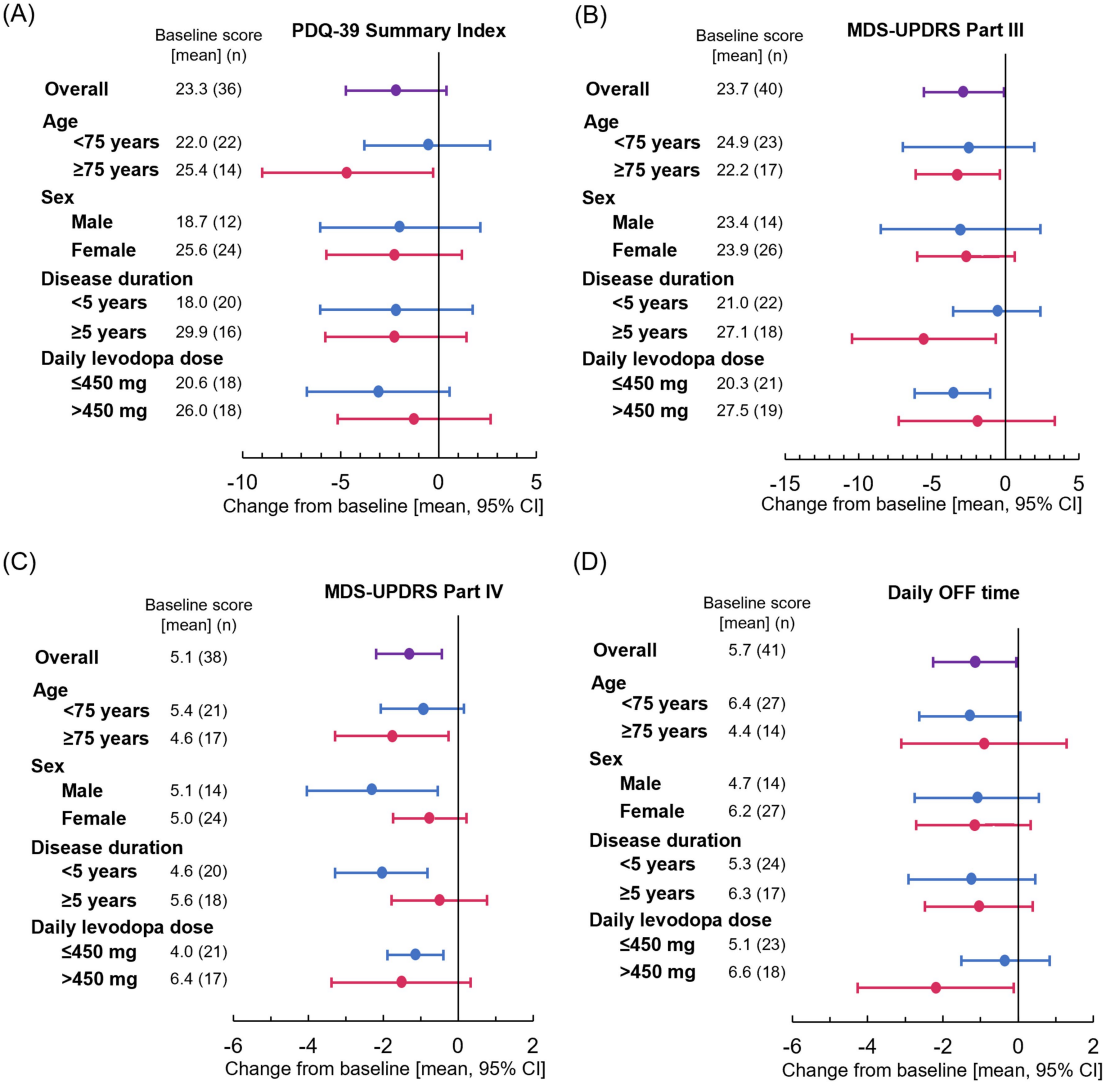
Effect of patient characteristics on change from baseline at Week 18 for **(A)** PDQ-39 Summary Index, **(B)** MDS-UPDRS Part III, **(C)** MDS-UPDRS Part IV, and **(D)** daily OFF time. Plots show means and error bars show 95% confidence intervals. The leftward trend indicates improvement. CI, confidence interval; MDS-UPDRS, Movement Disorder Society-Unified Parkinson’s Disease Rating Scale; PDQ-39, 39-item Parkinson’s Disease Questionnaire.

Although not statistically significant, the mean [SD] change in the PDQ-39 Summary Index exceeded the minimal clinical important difference (MCID) (−2.2 [7.5]; *p* = 0.094) (16). Significant improvements from baseline in mean [SD] change in MDS-UPDRS Part III and Part IV scores were also observed at Week 18 (−2.8 [8.5]; *p* = 0.043 and −1.3 [2.7]; *p* = 0.004, respectively). The mean [SD] change in daily OFF time at Week 18 from baseline was −1.2 [3.5] hours, representing a significant reduction from baseline (*p* = 0.041) (Figures 1A–D and Supplementary Table 1). Among the PDQ-39 sub-items, there was a statistically significant improvement in Mobility (mean [SD] change: −6.7 [13.6]; *p* = 0.004) and Activities of Daily Living (ADL) (−4.4 [12.2]; *p* = 0.031) subitems at Week 18 compared with baseline. Cognition and Bodily Discomfort subitems tended to be improved (mean [SD] change: −2.7 [13.3]; *p* = 0.218 and −5.5 [17.0]; *p* = 0.055, respectively), which were not statistical significant, but were both above the MCID (1.8 and 2.1, respectively). There was no difference in mean [SD] KPPS total score (0.0 [9.7]; *p* = 0.987) (Supplementary Table 2).

### 3.3 Subgroup analyses

Significant improvements in the PDQ-39 Summary Index were observed in patients aged ≥75 years (*p* = 0.039) (Figure 1A). Significant improvements in MDS-UPDRS Part III were also observed in patients aged ≥75 years (*p* = 0.029), those with a disease duration ≥5 years (*p* = 0.029), and those receiving levodopa dose ≤450 mg (*p* = 0.008) (Figure 1B). Significant improvements in MDS-UPDRS Part IV were observed in men (*p* = 0.014), patients aged ≥75 years (*p* = 0.025), those with a disease duration <5 years (*p* = 0.002), and those receiving levodopa dose ≤450 mg (*p* = 0.005) (Figure 1C). The point estimate of mean daily OFF time improved similarly regardless of sex, age, and disease duration (Figure 1D). The results of subgroup analysis are also shown in Supplementary Table 2.

#### 3.3.1 PDQ-39 subitems in subgroup analysis

**Figure 2:**
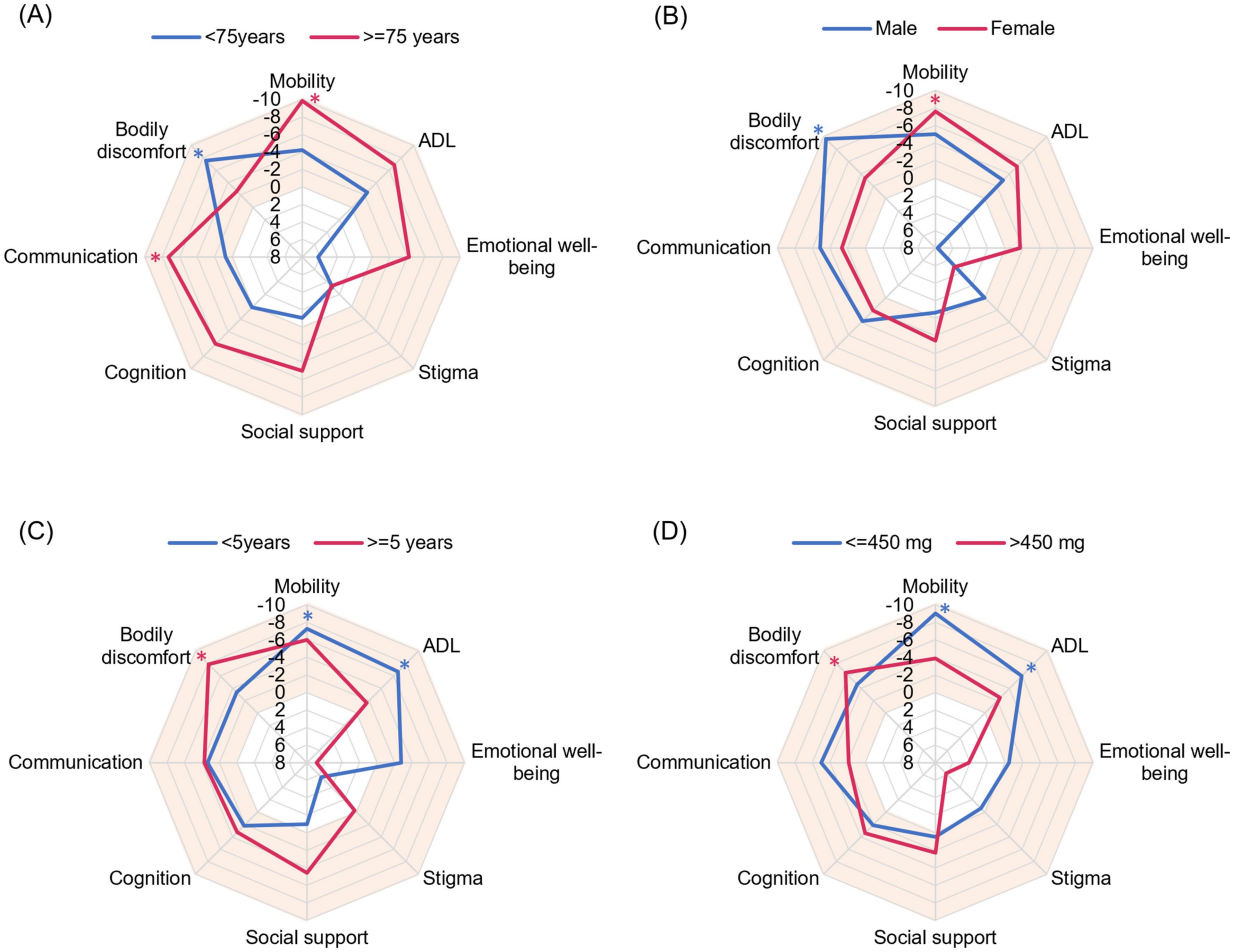
Radar plots evaluating the effect of patient characteristics on PDQ-39 Summary Index items. The mean change from baseline at Week 18 is plotted. A vertex in the orange area indicates improvement. The outward direction indicates greater improvement. **(A)** Age, **(B)** sex, **(C)** disease duration, and **(D)** daily levodopa dose at baseline. ADL, activities of daily living; PDQ-39, 39-item Parkinson’s Disease Questionnaire. *p < 0.05 compared with baseline.

When examined by age, patients aged ≥75 years experienced significant improvements in Mobility (*p* = 0.003) and Communication (*p* = 0.004), and patients aged <75 years experienced significant improvements in Bodily Discomfort (*p* = 0.025). ADL and Emotional well-being were more improved in patients aged ≥75 years than those aged <75 years (Figure 2A). When examined by sex, women experienced significant improvements in Mobility (*p* = 0.014) and men experienced significant improvements in Bodily Discomfort (*p* = 0.029) from baseline at Week 18. Women also experienced more improvement in ADL and Emotional well-being than men (Figure 2B). Patients with a disease duration <5 years and a levodopa dose ≤450 mg experienced significant improvements from baseline in Mobility (*p* = 0.035 and *p* = 0.001, respectively) and ADL (*p* = 0.035 and *p* = 0.046, respectively) at Week 18, and patients with a disease duration ≥5 years and levodopa dose >450 mg experienced significant improvements in Bodily Discomfort (*p* = 0.010 and *p* = 0.030, respectively). Emotional well-being tended to be better in patients with a disease duration <5 years and a levodopa dose ≤450 mg (*p* = 0.303 and *p* = 0.860, respectively) (Figures 2C,D). The results of subgroups analysis are also shown in Supplementary Table 2.

### 3.4 Path analysis

**Figure 3:**
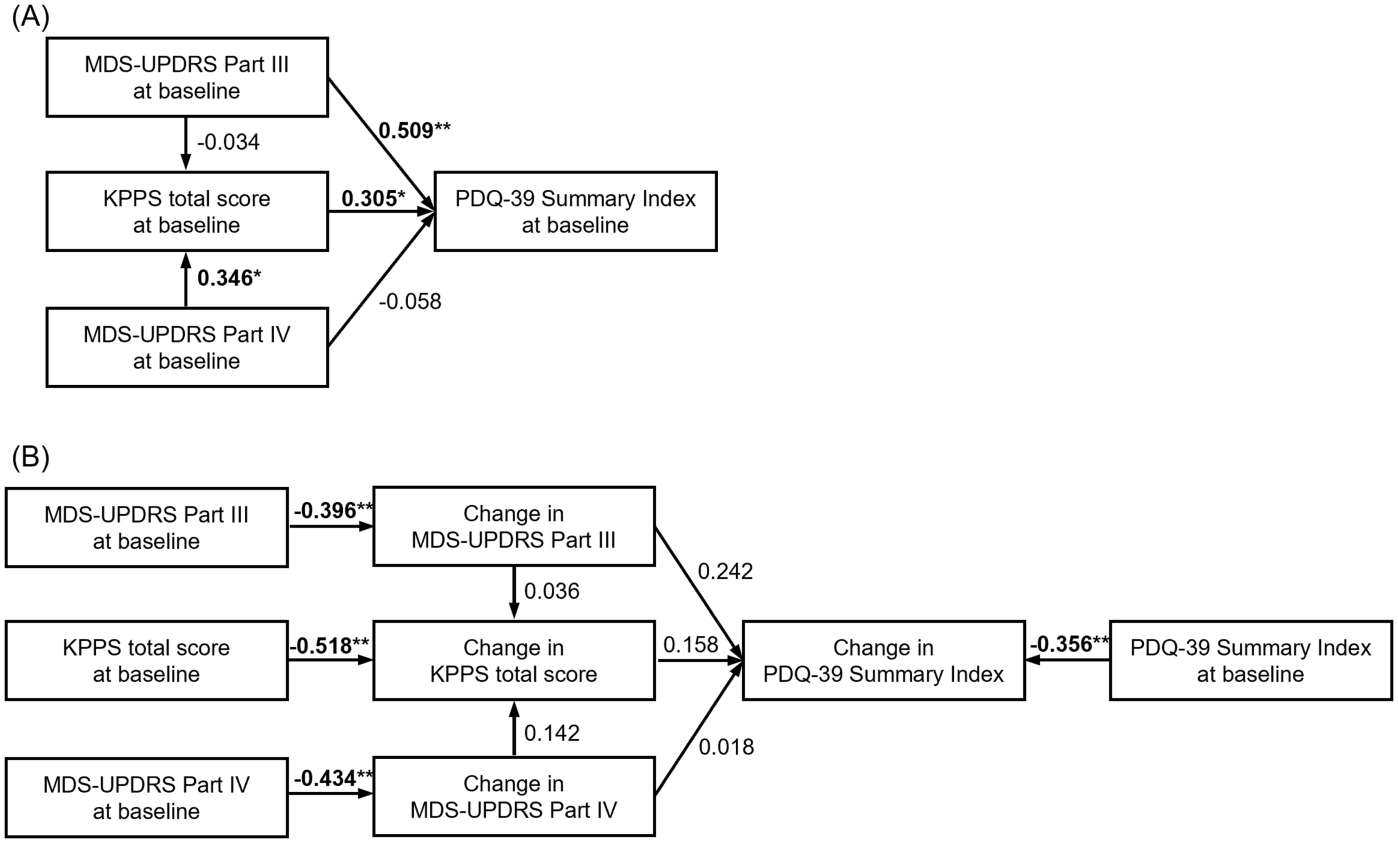
Path analysis investigating the relationship between variables at baseline and changes in the PDQ-39 Summary Index, MDS-UPDRS Part III and Part IV, and KPPS total score at Week 18. The number above the arrow represents the standardized path coefficient, which represents the effect of the variables at the start of the arrow on the variables at the end of the arrow. **(A)** Path analysis of variables at baseline (CFI = 1, RMSEA [90% CI] = 0 [0, 0]); **(B)** path analysis of variable change at baseline and at 18 weeks (CFI = 0.889, RMSEA [90% CI] = 0.083 [0.022, 0.177]). *p < 0.05, **p < 0.01. CI, confidence interval; CFI comparative fit index; KPPS, King’s Parkinson’s Pain Scale; MDS-UPDRS, Movement Disorder Society-Unified Parkinson’s Disease Rating Scale; PDQ-39, 39-item Parkinson’s Disease Questionnaire; RMSEA, root mean square error of approximation.

At baseline, the PDQ-39 Summary Index was significantly affected by motor symptoms (MDS-UPDRS Part III, path coefficient = 0.509, *p* < 0.001), which indicates a moderate effect, but not motor complications (MDS-UPDRS Part IV, path coefficient = −0.058, *p* = 0.671). Pain, as assessed by the KPPS total score, also significantly affected the PDQ-39 Summary Index (path coefficient = 0.305, *p* < 0.05), although to a lesser extent than motor symptoms, which indicates a weak effect (Figure 3A and Supplementary Table 3).

The change in each index at Week 18 was significantly affected by the baseline score, which means the worse the baseline score, the greater the improvement at Week 18 (*p* < 0.01 for all indexes). However, there was no statistically significant correlation between the changes in each index at Week 18. Changes in MDS-UPDRS Part III tended to affect the changes in the PDQ-39 Summary Index (path coefficient = 0.242, *p* = 0.089), which indicates a weak effect (Figure 3B and Supplementary Table 3).

## 4 Safety

AEs occurred in 24 patients (43.6%) and ADRs occurred in 12 patients (21.8%) (Supplementary Table 4). Discontinuations due to AEs occurred in 8 patients (14.5%), and due to ADRs in 7 patients (12.7%). The most common ADR was dyskinesia, of which there were 3 events (5.5%). One event (1.8%) each of anxiety disorder, blood bilirubin increased, dizziness, lack of drug efficacy, general physical health deterioration, hyperhidrosis, hypotension, myalgia, visual hallucination, and vomiting were reported, and there were no new safety concerns.

## 5 Discussion

The present pooled analysis showed that safinamide was well tolerated and improved motor symptoms and motor fluctuations and showed clinically meaningful improvement in QOL when added to levodopa monotherapy in patients with PD. The results of the subgroup analyses suggested that safinamide was effective, especially in patients aged 75 years or older, when it was used as add-on to levodopa monotherapy. The subgroup analysis for PDQ-39 subitems revealed the patient characteristics in which safinamide is more effective. Compared with previous clinical trials in Asia, our study included patients with a higher average age, which means that the patients in our study reflected those typically encountered in clinical practice (9, 10). In addition, the disease duration was approximately 3–5 years shorter than in the Asian clinical trial (9, 10), which means that patients with early-stage disease were included in our analysis. The mean daily levodopa dose at baseline was not very high, which also indicated that patients had early wearing-off in this study.

The PDQ-39 Summary Index also improved over the MCID (1.6), although there was no statistical difference compared with baseline (16). In addition, this result was consistent with a recent meta-analysis, which showed the efficacy of MAO-B inhibitors, especially safinamide, on QOL assessed by the PDQ-39 Summary Index (17). Mobility and ADL also improved significantly, and Cognition and Bodily Discomfort improved more than the MCID (1.8 and 2.1, respectively). Although the study design differed, these were the same subitems that improved in the post-hoc analysis of the SETTLE study conducted in Asian patients with PD, indicating the usefulness of safinamide in improving QOL (18). In the post-hoc analysis of Study 016 and the SETTLE study, the improvement in daily OFF time after 24 weeks in the levodopa monotherapy plus safinamide group was −1.59 h (placebo difference −1.35 h) (13). Although the evaluation period in the present study was 18 weeks, the improvement trend was similar to that of the previous report, with an improvement of −1.2 h. However, it requires noting that the result of the present study was in the single arm without a comparator. The Movement Disorder Society proposed that the levodopa equivalent daily dose of safinamide would be 150 mg (19). However, there are no direct comparisons between safinamide and levodopa regarding efficacy, and further investigation is needed to reveal whether adding safinamide to levodopa monotherapy or increasing the dose of levodopa is more effective.

In the subgroup analyses, the effect observed in the elderly group aged 75 years or older is noteworthy, with statistically significant improvements in PDQ-39 Summary Index (QOL), MDS-UPDRS Part III (motor symptoms), and MDS-UPDRS Part IV (motor complications). We considered it important to confirm the efficacy of safinamide as an adjunct to levodopa monotherapy in this study, while increasing the number of elderly patients treated with levodopa monotherapy for long-term (3). The reason for the high efficacy observed in the elderly population is thought to be related to MAO-B activity. MAO-B activity in the brain has been shown to increase with age in both healthy people and patients with PD (20, 21). Therefore, in the elderly patients with more active MAO-B, inhibition of MAO-B and elevation and stabilization of brain dopamine levels may have contributed to their improvement in symptoms.

In the exploratory path analysis to find factors influencing QOL, it was suggested that motor symptoms rather than motor complications affected QOL at baseline. A study conducted in Japan reported that daily OFF time affected QOL in patients with advanced PD but not in patients with non-advanced PD (22), which is consistent with the results of our analysis. In addition, the baseline MDS-UPDRS Part IV score of the patients in the current pooled analysis was 5.1, and motor complications were moderate (23), suggesting that the impact of motor complications on QOL was weaker than that of motor symptoms. Instead, pain at baseline had a statistically significant impact on QOL. A recent systematic review showed that motor symptoms affect QOL in patients with PD regardless of progression (24), and non-motor symptoms including pain have been shown to affect QOL in Japanese patients with PD (25). Therefore, the results of the path analysis were reasonable. In the other path analysis examining factors influencing the improvement in QOL, we observed that the worse a patient’s condition was at baseline, the greater the degree of improvement in MDS-UPDRS Parts III and IV and KPPS. Conversely, although no significant correlation was noted between changes in outcomes, improvement in MDS-UPDRS Part III tended to be associated with improvement in the PDQ-39 Summary Index. This result suggests that improvement in motor symptoms is important for improvement in QOL in patients receiving levodopa monotherapy. Non-motor symptoms are also important for patients with PD and wearing-off because they influence QOL (26). The path analysis to reveal the relationship between non-motor symptoms and QOL was not conducted due to the lack of evaluation of those symptoms in both studies. Further study is needed to analyze the relationship between non-motor symptoms and QOL.

In the subgroup analysis in terms of age, PDQ-39 Summary Index and MDS-UPDRS Part III and Part IV were improved in patients aged ≥75 years old. However, daily OFF time was not statistically improved in patients aged ≥75 years old, and the reduction in daily OFF time was greater in younger patients than older patients. Path analysis revealed that the improvement of MDS-UPDRS Part III and Part IV tended to contribute to the improvement of PDQ-39 Summary Index. Older patients experienced greater improvements in MDS-UPDRS Parts III and IV compared with younger patients; therefore older patients also experienced a greater improvement in the PDQ-39 Summary Index, which was consistent with the result of path analysis. However, in terms of daily OFF time, the accuracy of the symptom diary has been questioned in some recent papers (27, 28). Therefore, it is possible that patients could not evaluate their motor fluctuation precisely in this study. The previous report showed that the digital tool is helpful for accurately assessing PD symptoms (29), so further study might be needed using wearable devices or applications.

In terms of PDQ-39 subitems, Mobility, ADL, and Emotional Well-Being were more greatly improved in patients who were female and 75 years or older and had a shorter disease duration and lower levodopa dose at baseline. In contrast, Bodily Discomfort was improved in patients who were male and <75 years and had a longer disease duration and higher levodopa dose at baseline. These results suggest that safinamide efficacy differed according to patient background characteristics. In the Japanese phase 3 trial of safinamide, remarkable improvements in UPDRS Part III (motor symptoms) and Part II (ADL) were observed (9). In contrast, the international phase 3 trial showed that safinamide significantly improved Bodily Discomfort in PDQ-39 (18). The difference between the study populations in these clinical trials is that the Japanese trial had fewer male patients and a lower dose of levodopa (male: 41.9–49.2% vs. 59.3–62.4%, mean levodopa dose: 420–450 mg/day vs. 760–790 mg/day) (7, 9). The characteristics of safinamide’s effectiveness on PDQ-39 sub items found in the subgroup analyses appear to be consistent given the study populations in these clinical trials (30). Safinamide completely (90%) inhibits MAO-B activity at the dose of 50 mg in humans (31). In addition, safinamide inhibits neuronal voltage-gated sodium and calcium channels and the subsequent release of glutamate. An animal study suggests that the effect of safinamide on pain is dose-dependent and may be exerted at higher doses (32). Improvement of motor symptoms and ADL with safinamide can be explained by dopaminergic action, and improvement of Bodily Discomfort can be explained by sodium channel inhibition (33). Further studies are needed to confirm the relationship between the efficacy and mode of action.

With respect to safety, AEs and ADRs occurred in 43.6 and 21.8% of patients, respectively, which is comparable to the results of post-marketing studies conducted in Europe (34). Dyskinesia occurred in three patients (5.5%), which is similar to the rate reported in the post-marketing surveillance of rasagiline, which included 40% of patients with early PD who did not develop wearing-off (35). The use of safinamide in patients with early PD treated with levodopa alone and experiencing wearing-off was confirmed to be tolerable.

There are some limitations in the present study. Firstly, this analysis utilized a single arm of the J-SILVER and KEEP studies, thus this was in essence a single-arm study with no randomization, and the efficacy results may include placebo effects. However, the results of this analysis can provide useful information for predicting the effects of safinamide in real-world clinical practice. Secondly, only 54 patients were included in the FAS, 41 of whom completed the study, and a large-scale study is required to verify our findings. Thirdly, the study duration was also only 18 weeks, and only short-term efficacy and safety were investigated in this study. Additional studies are therefore required to evaluate the long-term efficacy and safety of safinamide and levodopa. Fourthly, in the subgroup analyses, the results regarding wearing-off were not consistent with other outcomes. The low accuracy of the symptom diary might be one reason, because these studies were conducted under daily clinical practice rather than well-managed clinical trials. Regarding the path analysis, contributions from other factors cannot be ruled out, especially non-motor symptoms in PD. Finally, the J-SILVER and KEEP studies also differed with respect to the safinamide dose schedule and the patient selection criteria, and the results should be interpreted with caution. In particular, it is difficult to investigate the efficacy of increasing the dose to 100 mg/day from this study, and further studies are anticipated.

## 6 Conclusion

In conclusion, the addition of safinamide to levodopa monotherapy improved motor symptoms, motor complications, and QOL without any new tolerability concerns. The present study showed that safinamide as an adjunct to levodopa monotherapy is efficacious, particularly in elderly patients aged 75 years or older. Path analysis revealed that the QOL of patients with PD who are treated with levodopa monotherapy is largely influenced by motor symptoms, not motor complications. These results suggested that it is important to improve motor symptoms from the viewpoint of improving QOL in early-stage wearing-off in patients with PD.

## Data Availability

The data that support the findings of this study are not openly available due to confidentiality reasons but are available from the corresponding author upon reasonable request.
