# Supplementary material for: Safinamide as an adjunct to levodopa monotherapy in Asian patients with Parkinson’s disease experiencing early wearing-off: a pooled analysis of the J-SILVER and KEEP studies
Source: Front Neurol. 2025 Jun 2;16:1591664. doi: 10.3389/fneur.2025.1591664 (PMC12171447; doi:10.3389/fneur.2025.1591664)
Supplement: Supplementary file 1 [file Supplementary_file_1.docx]

Supplementary Material

# Supplementary Figures and Tables

## Supplementary Figure

**Supplementary Figure 1.** Patient flow diagram.


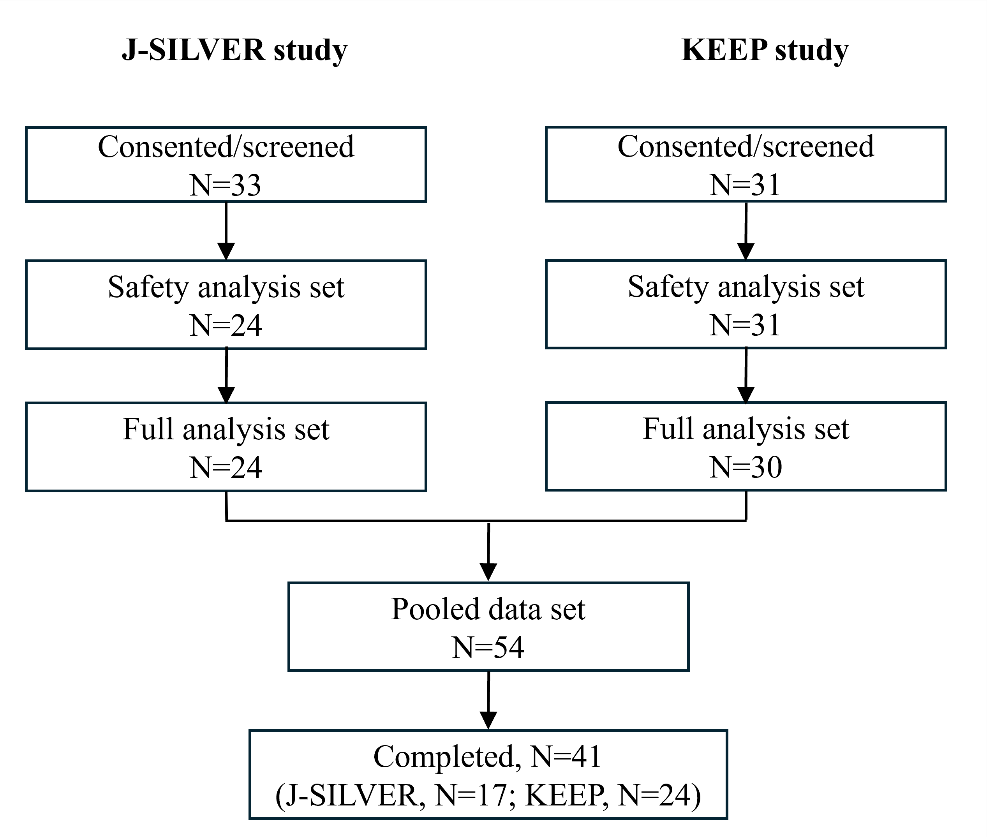


## Supplementary Tables

**Supplementary Table 1.** Change from baseline at Week 18 for PDQ-39

| Efficacy Outcome | N | Baseline value | Mean (SD) change from baseline at Week 18 | P-value | Minimally important difference |
| --- | --- | --- | --- | --- | --- |
| PDQ-39 Summary Index | 36 | 23.3 (13.0) | -2.2 (7.5) | 0.094 | 1.6 |
| 1. Mobility | 39 | 33.6 (23.0) | -6.7 (13.6) | **< 0.01** | 3.2 |
| 2. ADL | 39 | 23.5 (21.4) | -4.4 (12.2) | **< 0.05** | 4.4 |
| 3. Emotional well-being | 39 | 23.6 (18.2) | 1.7 (20.1) | 0.598 | 4.2 |
| 4. Stigma | 39 | 21.2 (22.3) | 3.2 (22.4) | 0.378 | 5.6 |
| 5. Social support | 37 | 12.6 (15.4) | -1.4 (10.6) | 0.424 | 11.4 |
| 6. Cognition | 39 | 27.7 (20.5) | -2.7 (13.3) | 0.218 | 1.8 |
| 7. Communication | 38 | 16.0 (21.2) | -3.5 (14.6) | 0.146 | 4.2 |
| 8. Bodily discomfort | 38 | 25.9 (18.0) | -5.5 (17.0) | 0.055 | 2.1 |

Bold is statistically significant (p<0.05).

ADL, activities of daily living; PDQ-39, 39-item Parkinson's Disease Questionnaire; SD, standard deviation.

**Supplementary Table 2.** The results of the subgroup analyses

|  | Overall | | Age | | | | Sex | | | | Disease duration | | | | Daily levodopa dose at baseline | | | |
| --- | --- | --- | --- | --- | --- | --- | --- | --- | --- | --- | --- | --- | --- | --- | --- | --- | --- | --- |
|  |  |  | <75 years | | ≥75 years | | Male | | Female | | <5 years | | ≥5 years | | ≤450 mg | | >450 mg | |
|  | BL | CFB | BL | CFB | BL | CFB | BL | CFB | BL | CFB | BL | CFB | BL | CFB | BL | CFB | BL | CFB |
| PDQ-39 Summary Index | 23.3 (13.0) | -2.2  (-4.7, 0.4) | 22.0 (11.8) | -0.6  (-3.8, 2.6) | 25.4 (14.8) | **-4.6**  (-9.0,  -0.3) | 18.7 (9.2) | -2.0  (-6.0, 2.1) | 25.6 (14.1) | -2.3  (-5.7, 1.2) | 18.0 (10.2) | -2.2  (-6.0, 1.7) | 29.9 (13.3) | -2.2  (-5.8, 1.4) | 20.6 (13.3) | -3.1  (-6.7, 0.6) | 26.0 (12.4) | -1.3 (-5.2, 2.7) |
| 1. Mobility | 33.6 (23.0) | **-6.7**  (-11.1, 　-2.3) | 30.6 (22.9) | -4.2  (-10.7, 2.3) | 37.5 (23.3) | **-9.9**  (-15.9, -3.8) | 27.7 (23.7) | -5.0  (-12.2, 2.2) | 36.9 (22.4) | **-7.6**  (-13.5, -1.7) | 27.0 (21.1) | **-7.3**  (-14.0, -0.6) | 41.3 (23.3) | -6.0  (-12.2, 0.3) | 26.1 (20.2) | **-9.0** (-14.1,　 -4.0) | 42.4 (23.5) | -3.9 (-11.8, 4.0) |
| 2. ADL | 23.5 (21.4) | **-4.4**  (-8.4, 　-0.4) | 24.4 (21.8) | -2.5  (-6.4, 1.5) | 22.3 (21.5) | -6.9  (-14.8, 1.1) | 25.9 (17.8) | -3.0  (-7.8, 1.9) | 22.2 (23.4) | -5.2  (-11.0, 0.6) | 21.6 (23.1) | **-6.7**  (-13.0, -0.5) | 25.7 (19.6) | -1.6  (-6.6, 3.3) | 21.4 (23.7) | **-6.0**  (-11.8, -0.1) | 25.9 (18.8) | -2.5 (-8.3, 3.2) |
| 3. Emotional well-being | 23.6 (18.2) | 1.7  (-4.8, 8.2) | 21.4 (18.8) | 6.2  (-4.6, 17.1) | 26.5 (17.5) | -4.2  (-9.7, 1.4) | 11.0 (7.4) | 7.7  (-8.2, 23.7) | 30.7 (18.7) | -1.7  (-7.4, 4.1) | 18.1 (12.1) | -2.8  (-8.3, 2.7) | 30.1 (22.0) | 6.9  (-6.0, 19.9) | 19.2 (15.2) | -0.4  (-5.0, 4.3) | 28.7 (20.4) | 4.2  (-9.6, 18.0) |
| 4. Stigma | 21.2 (22.3) | 3.2  (-4.1, 10.5) | 24.4 (23.9) | 3.1  (-7.4, 13.6) | 16.9 (20.1) | 3.3  (-7.7, 14.3) | 12.5 (19.0) | 0.0  (-6.6, 6.6) | 26.0 (22.9) | 5.0  (-6.1, 16.1) | 14.0 (17.2) | 5.7  (-2.8, 14.1) | 29.5 (25.1) | 0.3  (-12.8, 13.5) | 21.1 (23.4) | 0.6  (-9.8, 11.0) | 21.2 (21.7) | 6.3  (-4.8, 17.3) |
| 5. Social support | 12.6 (15.4) | -1.4  (-4.9, 2.1) | 10.9 (12.5) | 1.0  (-3.6, 5.7) | 15.1 (19.0) | -5.0  (-10.5, 0.5) | 5.3 (9.5) | 0.6  (-5.1, 6.4) | 16.6 (16.6) | -2.5  (-7.2, 2.2) | 6.0 (7.2) | 1.0  (-3.3, 5.3) | 21.4 (18.9) | -4.6  (-10.7, 1.6) | 7.8 (13.1) | -0.5  (-3.6, 2.5) | 17.7 (16.3) | -2.3 (-9.2, 4.6) |
| 6. Cognition | 27.7 (20.5) | -2.7  (-7.0, 1.7) | 24.2 (20.8) | -0.1  (-6.2, 6.0) | 32.2 (19.8) | -6.0  (-12.4, 0.4) | 24.1 (18.5) | -3.7  (-8.6, 1.1) | 29.8 (21.7) | -2.1  (-8.5, 4.3) | 26.3 (19.4) | -2.2  (-8.2, 3.8) | 29.4 (22.2) | -3.2  (-10.1, 3.6) | 28.2 (21.2) | -2.1  (-6.2, 2.1) | 27.2 (20.3) | -3.4 (-12.0, 5.3) |
| 7. Communication | 16.0 (21.2) | -3.5  (-8.3, 1.3) | 15.9 (21.8) | -0.8  (-8.5, 7.0) | 16.1 (21.0) | **-7.3**  (-11.8, -2.7) | 15.4 (20.1) | -5.1  (-17.0, 6.8) | 16.3 (22.1) | -2.7 (-7.4, 2.1) | 10.8 (17.1) | -3.3  (-11.2, 4.5) | 21.8 (24.1) | -3.7  (-9.8, 2.4) | 14.2 (19.7) | -5.0  (-12.0, 2.0) | 18.1 (23.1) | -1.9 (-9.0, 5.3) |
| 8. Bodily discomfort | 25.9 (18.0) | -5.5 (-11.1, 0.1) | 24.2 (18.7) | **-7.6**  (-14.1, -1.0) | 28.1 (17.4) | -2.6  (-13.2, 8.0) | 22.4 (14.2) | **-9.6**  (-18.1, -1.2) | 27.7 (19.8) | -3.3  (-10.9, 4.2) | 20.8 (13.1) | -3.3  (-13.1, 6.4) | 31.5 (21.3) | **-7.9**  (-13.6, -2.1) | 25.0 (19.9) | -4.6  (-14.4, 5.3) | 26.9 (16.3) | **-6.5** (-12.3, -0.7) |
| Daily OFF time (hours) | 5.7 (3.3) | **-1.2**  (-2.3,　 -0.1) | 6.4 (3.3) | -1.3  (-2.6, 0.1) | 4.4 (3.1) | -0.9  (-3.1, 1.3) | 4.7 (3.0) | -1.1  (-2.8, 0.6) | 6.2 (3.4) | -1.2  (-2.7, 0.3) | 5.3 (3.6) | -1.2  (-2.9, 0.5) | 6.3 (2.8) | -1.0  (-2.5, 0.4) | 5.1 (2.6) | -0.3  (-1.5, 0.8) | 6.6 (3.9) | **-2.2** (-4.3, -0.1) |
| MDS-UPDRS Part III total score | 23.7 (11.2) | **-2.8**  (-5.6,  -0.1) | 24.9 (13.3) | -2.5  (-7.0, 1.9) | 22.2 (7.6) | **-3.2**  (-6.1,  -0.4) | 23.4 (13.2) | -3.1  (-8.5, 2.4) | 23.9 (10.2) | -2.7  (-6.0, 0.6) | 21.0 (12.0) | -0.6  (-3.6, 2.4) | 27.1 (9.3) | **-5.6**  (-10.5, -0.7) | 20.3 (11.2) | **-3.6**  (-6.2,  -1.1) | 27.5 (10.1) | -1.9 (-7.3, 3.4) |
| MDS-UPDRS Part IV total score | 5.1 (2.1) | **-1.3**  (-2.2,  -0.4) | 5.4 (2.1) | -1.0  (-2.1, 0.2) | 4.6 (2.2) | **-1.8**  (-3.3, 　-0.3) | 5.1 (2.4) | **-2.3**  (-4.0,  -0.5) | 5.0 (2.0) | -0.8  (-1.7, 0.2) | 4.6 (2.2) | **-2.1**  (-3.3,  -0.8) | 5.6 (2.1) | -0.5  (-1.8, 0.8) | 4.0 (1.8) | **-1.1**  (-1.9, 　-0.4) | 6.4 (1.8) | -1.5 (-3.4, 0.3) |
| KPPS total score | 6.8 (8.4) | 0.0  (-3.1, 3.2) | 6.0 (10.1) | 1.0  (-4.0, 5.9) | 7.9 (5.5) | -1.2  (-5.0, 2.7) | 5.8 (4.6) | 2.1  (-5.3, 9.4) | 7.4 (9.9) | -1.1  (-4.2, 2.0) | 5.6 (4.7) | -0.9  (-3.8, 2.1) | 8.3 (11.2) | 1.1  (-5.2, 7.3) | 4.6 (3.8) | 0.9  (-1.3, 3.2) | 9.4 (11.2) | -1.0  (-7.6, 5.6) |

Bold is statistically significant (p<0.05).

BL, Baseline: mean (SD); CFB, change from baseline [95% CI].

ADL, activities of daily living; CI, confidence interval; KPPS; King's Parkinson's Pain Scale; MDS-UPDRS, Movement Disorder Society-Unified Parkinson's Disease Rating Scale; PDQ-39, 39-item Parkinson's Disease Questionnaire; SD, standard deviation.

**Supplementary Table 3.** Results of path analysis

| At Baseline | | | |
| --- | --- | --- | --- |
| Endogenous variables | Exogenous variables | Path coefficient  [95%CI] | P-value |
| Baseline PDQ-39 Summary Index | Baseline MDS-UPDRS part III | 0.509 [0.294, 0.723] | <0.001 |
|  | Baseline KPPS total score | 0.305 [0.070, 0.540] | 0.011 |
|  | Baseline MDS-UPDRS part IV | -0.058 [-0.328, 0.211] | 0.671 |
| Baseline KPPS total score | Baseline MDS-UPDRS part III | -0.034 [-0.341, 0.274] | 0.829 |
|  | Baseline MDS-UPDRS part IV | 0.346 [0.064, 0.628] | 0.016 |
| Model fit measures | | CFI=1 | |
|  |  | RMSEA [90% CI] =0 [0, 0] | |
| At Week 18 | | | |
| Endogenous variables | Exogenous variables | Path coefficient  [95%CI] | P-value |
| Change in PDQ-39 Summary Index | Change in MDS-UPDRS part III | 0.242 [-0.037, 0.520] | 0.089 |
|  | Change in KPPS total score | 0.158 [-0.102, 0.418] | 0.234 |
|  | Change in MDS-UPDRS part IV | 0.018 [-0.264, 0.299] | 0.903 |
|  | Baseline PDQ-39 Summary Index | -0.356 [-0.586, -0.126] | 0.002 |
| Change in MDS-UPDRS part III | Baseline MDS-UPDRS part III | -0.396 [-0.586, -0.126] | 0.001 |
| Change in KPPS total score | Change in MDS-UPDRS part III | 0.036 [-0.207, 0.279] | 0.774 |
|  | Change in MDS-UPDRS part IV | 0.142 [-0.102, 0.387] | 0.254 |
|  | Baseline KPPS total score | -0.518 [-0.723, -0.313] | <0.001 |
| Change in MDS-UPDRS part IV | Baseline MDS-UPDRS part IV | -0.434 [-0.660, -0.208] | <0.001 |
| Model fit measures | | CFI=0.889 | |
|  |  | RMSEA [90% CI] =0.083 [0.022, 0.177] | |

CI, confidence interval; CFI, comparative fit index; KPPS; King's Parkinson's Pain Scale; MDS-UPDRS, Movement Disorder Society-Unified Parkinson's Disease Rating Scale; PDQ-39, 39-item Parkinson's Disease Questionnaire; RMSEA, root mean square error of approximation.

**Supplementary Table 4.** Adverse events and adverse drug reactions during the 18-week study period

|  | AE | ADR |
| --- | --- | --- |
| All events, n (%)^1^ | 24 (43.6) | 12 (21.8) |
| Discontinuation due to AE/ADR, n (%)^1^ | 8 (14.5) | 7 (12.7) |
| AEs/ADRs, n (%)^2^ |  |  |
| Dyskinesia | 3 (5.5) | 3 (5.5) |
| Headache | 2 (3.6) | 0 (0.0) |
| Overdose | 2 (3.6) | 0 (0.0) |
| Wrist fracture | 2 (3.6) | 0 (0.0) |
| Anxiety disorder | 1 (1.8) | 1 (1.8) |
| Blood bilirubin increased | 1 (1.8) | 1 (1.8) |
| Constipation | 1 (1.8) | 0 (0.0) |
| COVID-19 | 1 (1.8) | 0 (0.0) |
| Delirium | 1 (1.8) | 0 (0.0) |
| Dizziness | 1 (1.8) | 1 (1.8) |
| Drug ineffective | 1 (1.8) | 1 (1.8) |
| Fatigue | 1 (1.8) | 0 (0.0) |
| General physical health deterioration | 1 (1.8) | 1 (1.8) |
| Hyperhidrosis | 1 (1.8) | 1 (1.8) |
| Hypotension | 1 (1.8) | 1 (1.8) |
| Lumbar spinal canal stenosis | 1 (1.8) | 0 (0.0) |
| Myalgia | 1 (1.8) | 1 (1.8) |
| Nasopharyngitis | 1 (1.8) | 0 (0.0) |
| Nausea | 1 (1.8) | 0 (0.0) |
| RBD | 1 (1.8) | 0 (0.0) |
| Tooth fracture | 1 (1.8) | 0 (0.0) |
| Visual hallucination | 1 (1.8) | 1 (1.8) |
| Vomiting | 1 (1.8) | 1 (1.8) |

^1^Number of patients

^2^Number of events

ADR, adverse drug reaction; AE, adverse event; COVID-19, coronavirus disease of 2019; RBD, rapid eye movement sleep behavior disorder.

**Supplementary Table 5.** The list of study sites

| Site No. | Site Name |
| --- | --- |
| Japan (J-SILVER study) | |
| 1 | Faculty of Medicine, Juntendo University |
| 2 | Juntendo University Nerima Hospital |
| 3 | Juntendo University Koshigaya Hospital |
| 4 | Juntendo University Urayasu Hospital |
| 5 | Juntendo Tokyo Koto Geriatric Medical Center |
| Republic of Korea (KEEP study) | |
| 1 | Inje University Sanggye Paik Hospital |
| 2 | Kyungpook National University Chilgok Hospital |
| 3 | Inje University Busan Paik Hospital |
| 4 | Kyung Hee University Hospital |
| 5 | Korea University Ansan Hospital |
| 6 | Korea University Guro Hospital |
| 7 | Seoul St. Mary’s Hospital |
| 8 | Samsung Medical Center |
| 9 | Yonsei University Health System Severance Hospital |
| 10 | Chonnam National University Hospital |
| 11 | Dong-A University Hospital |
| 12 | Soon Chun Hyang University Hospital Bucheon |
| 13 | Chung-Ang University Hospital |
| 14 | Gachon University Gil Medical Center |
| 15 | Hallym University Sacred Heart Hospital |
| 16 | Yeungnam University Medical Center |
| 17 | Seoul National University Bundang Hospital |
| 18 | Gangnam Severance Hospital |
| 19 | Chungnam National University Hospital |
| 20 | Inje University Haeundae Paik Hospital |

## Supplementary Methods - Statistical Methodology used in the Path Analysis

- - In order to examine the relationship between the baseline variables, the following model was assumed, in which the endogenous variables were the actual baseline values of the PDQ-39 Summary Index (pdq_b) and the KPPS total score (kpps_b), and the exogenous variables were the actual baseline values of the MDS-UPDRS part III (upd3_b) and the MDS-UPDRS part IV (upd4_b).

$$pdq\_b\sim upd3\_b+kpps\_b+upd4\_b+pdq\_b$$

$$kpps\_b\sim upd3\_b+upd4\_b$$

- - In order to investigate the relationship between changes at Week 18, the following model was assumed, in which the endogenous variables were the change from baseline at Week 18 in the PDQ-39 Summary Index (pdq_c), the change from baseline at Week 18 in the MDS-UPDRS part III (upd3_c), the change from baseline at Week 18 in the KPPS total score (kpps_c), and the change from baseline at Week 18 in the MDS-UPDRS part IV (upd4_c), and the exogenous variables were the actual baseline measurement in the MDS-UPDRS part III, the actual baseline measurement in the KPPS total score, the actual baseline measurement in the MDS-UPDRS part IV, and the actual baseline measurement in the PDQ-39 Summary Index.

$$pdq\_c\sim upd3\_c+kpps\_c+upd4\_c+pdq\_b$$

$$upd3\_c\sim upd3\_b$$

$$kpps\_c\sim upd3\_c+upd4\_c+kpps\_b$$

$$upd4\_c\sim upd4\_$$
